# The Impact of Caring on Psychological Wellbeing: A Qualitative Study in Carers of People Living With Behavioural-Variant Frontotemporal Dementia

**DOI:** 10.1177/08919887251401262

**Published:** 2025-11-21

**Authors:** Isabella M. Ocampo, Jemma Todd, Grace Wei, Fiona Kumfor

**Affiliations:** 1 The University of Sydney, School of Psychology and Brain and Mind Centre, Camperdown, NSW, Australia; 2 University of Western Australia, School of Psychological Science, Perth, WA, Australia

**Keywords:** behavioural-variant frontotemporal dementia, carers, carer support, lived experience, mental health, psychological wellbeing

## Abstract

**Background:** Informal carers of people living with dementia can experience increased burden, stress and a decline in mental health and wellbeing. Growing evidence suggests these impacts are greater for those caring for individuals living with behavioural-variant frontotemporal dementia. However, little is known about their psychological needs and how these carers access psychological support. **Objective: **This study aimed to understand the impact of caring on psychological wellbeing and the experiences and needs of carers in seeking psychological support. **Methods:** Twelve carers participated in either a group or individual semi-structured interview. Data were analysed qualitatively using thematic analysis. **Results:** Five themes emerged: (1) *The job of being a carer*, (2) *The carer and person living with bvFTD as a unit*: *Reciprocal wellbeing & Care for the carers*, (3) *Feeling seen, Feeling heard, and Being connected*, (4) *The power of information* and (5) *Meaning and purpose through caring*. These themes captured the challenges, supports and meaningful aspects of the caring experience, and their impact on psychological wellbeing. While carers reported both positive and negative impacts of caregiving on their psychological wellbeing, they also highlighted a lack of tailored services in the context of behavioural-variant frontotemporal dementia. **Conclusions:** Psychological support services for carers should address common barriers to accessibility and flexibility and be developed in collaboration with carers to optimise feasibility and efficacy.

## Introduction

Frontotemporal dementia refers to a group of neurodegenerative syndromes characterized by progressive decline in behaviour and/or language due to atrophy in the frontal and/or temporal lobes.^1,2^ It is one of the most common forms of younger-onset dementia, with a prevalence rate of 13/100 000 in the 40-64 age range.^3^ Of its three primary variants; behavioural-variant, semantic variant primary progressive aphasia, and non-fluent variant primary progressive aphasia, behavioural-variant frontotemporal dementia has the youngest peak age at diagnosis, at 60-64 years^3^ and is estimated to be four times more common than the other variants.^4^ The features most prominent in behavioural-variant frontotemporal dementia include changes in behaviour and personality and a progressive decline in social functioning and self-regulation.^2^ These behavioural-variant frontotemporal dementia symptoms have an insidious onset and are recognised to overlap with psychiatric symptoms.^5^ Furthermore, the person living with behavioural-variant frontotemporal dementia often has limited insight,^2,6^ leaving family members with greater responsibility over the care and management of the disease.

Significant carer burden has been identified in samples of mixed frontotemporal dementia variants, with carers reporting greater overall burden^7,8^ and distress related to behavioural disturbances,^9^ than carers of people living with Alzheimer’s disease. In addition, some carers in mixed frontotemporal dementia samples report clinically significant levels of depression, anxiety, strain, and stress.^7,10,11^ Average levels of these outcomes have been found to be greater in carers of people living with behavioural-variant frontotemporal dementia compared to those caring for people living with Alzheimer’s disease and other frontotemporal dementia variants.^7,12,13^

The causes of this increased carer burden and psychological distress are multifactorial. In studies examining frontotemporal dementia more broadly, the diagnostic delay can be stressful, confusing, and a time of uncertainty for carers.^14-16^ At the time of diagnosis, the person living with frontotemporal dementia and their family members are often still working, leading to additional financial strain.^10,12,17^ The person with frontotemporal dementia may also have children living at home, who then experience the behaviour and personality changes more directly.^18-20^ Finally, the behavioural symptoms in frontotemporal dementia are frequently reported as the most burdensome for carers and have been associated with negative carer outcomes.^6,7,9,16,21,22^ However, this research has been limited primarily to depression, anxiety or stress as assessed through quantitative measures. Here, we elected to use qualitative methods to provide a rich, nuanced understanding of the broader impacts on carers–such as psychological wellbeing and mental health–or the contexts in which these experiences occur, and without limiting carers to predefined mental health outcomes or symptoms. Importantly, most studies, including a few using qualitative methods,^18,19,22,23^ do not distinguish between frontotemporal dementia variants, providing limited insight into the unique experience of carers of people living with behavioural-variant frontotemporal dementia.

Because of the lower prevalence and awareness of behavioural-variant frontotemporal dementia than other dementias like Alzheimer’s disease, specialised supports for carers of people living with behavioural-variant frontotemporal dementia are lacking.^10,12^ Informal carer interventions such as carer support groups are often developed for Alzheimer’s disease or dementia in general; these fail to address challenges unique to behavioural-variant frontotemporal dementia^16,22,24^ and contribute to carers feeling isolated and unsupported.^15,25^ Indeed, carers in mixed frontotemporal dementia samples have reported developing their own coping strategies such as: seeking information and practical strategies to manage frontotemporal dementia symptoms, seeking professional and social support, and joining support groups.^15,17,23^ However, only one previous study explored the experience of carers of people living with behavioural-variant frontotemporal dementia exclusively,^15^ and none explicitly examined carers’ experiences accessing services for their own mental health and psychological wellbeing. Given previous findings of increased carer burden and lack of carer support in the context of behavioural-variant frontotemporal dementia, better understanding of the needs of this population is necessary.

One possible avenue for carer support is the development of formal psychological interventions, such as cognitive-behavioural therapy, mindfulness-based therapies or acceptance-based therapies. These types of psychological interventions have been developed and examined for use in Alzheimer’s disease or dementia more broadly, yielding small to large benefits on carer burden, stress, anxiety, and depression.^25-28^ However, to our knowledge psychological interventions have not been developed specifically for carers of people living with behavioural-variant frontotemporal dementia. Furthermore, the extent to which carers of people living with behavioural-variant frontotemporal dementia access formal psychological support and perceive it as suitable has not been examined. Adopting a bottom-up, user-driven approach represents an important first step towards understanding the experiences and needs of carers of people living with behavioural-variant frontotemporal dementia in accessing support for their mental health and psychological needs.

This study aims to: (i) understand how caring for someone living with behavioural-variant frontotemporal dementia can impact mental health and psychological wellbeing; (ii) identify the supports that carers of people living with behavioural-variant frontotemporal dementia have accessed and (iii) examine carers’ wants or needs in terms of support for their mental health and psychological wellbeing. In addition, it explored carers’ experiences and attitudes towards accessing formal psychological support, such as clinical psychology services. Given the exploratory nature of its aims, the study employed a qualitative approach using group and individual interviews. Qualitative approaches enable a thorough, participant-led exploration of lived experience, and allows for the richness and complexity of participants’ perspectives to emerge.^29-31^

## Method

### Study Context and Setting

This study was conducted in Sydney, Australia, where individuals typically have access to a range of government-funded and private support systems. These include the National Disability Insurance Scheme (NDIS) for individuals under 65 living with a disability, Centrelink for financial assistance, and My Aged Care for aged care services. Public and private hospitals or clinics may offer community or in-home services such as nursing, allied health, respite care, and palliative care, while not-for-profit dementia organisations (e.g. Dementia Australia) and research clinics can offer information and services to carers and people living with dementia. Psychology and counselling may be available through these organisations, albeit often short-term, or through formal referrals to private practices.

### Participants

Participants were recruited from the FRONTIER Frontotemporal Dementia Research Clinic at the Brain and Mind Centre, a publicly-funded, university-based specialist research clinic. Individuals were eligible for participation if they spoke English, were over 18 years of age and were involved in the care of someone living with behavioural-variant frontotemporal dementia, hereafter referred to as “person living with bvFTD”. A member of FRONTIER, independent from the research team, identified eligible participants from a database of individuals who have consented to being re-contacted for future research. The FRONTIER member identified eligible participants with consideration for diversity in carer experiences (e.g. relationship to the person living with bvFTD, living situation, and stage in the carer trajectory-including bereavement). Individuals with acute or serious mental health difficulties, for whom participation in the study may be distressing, were not identified as eligible.

Twelve potential participants were initially identified and sent invitation emails. Invitation emails were sent to an additional 20 eligible participants until data reached saturation, such that a range of ideas were identified, and no new ideas emerged. Seventeen individuals responded to the invitation email and were provided written information on the study. Two individuals withdrew their interest, and 3 were not available to participate; no further data was collected from these individuals.

The study was approved by the University of Sydney Human Research Ethics Committee (2022/429). Twelve individuals provided verbal and written consent to participate in accordance with the Declaration of Helsinki and its later amendments. Each participant was phoned to collect basic demographic information and arrange a time for a group interview. In cases where a participant was uncomfortable to participate in a group setting or unable to attend a scheduled group interview, individual semi-structured interviews were conducted.

### Interviews

Four 90-minute group interviews, each with 2-3 participants, and two 60-minute individual semi-structured interviews were conducted and recorded via Zoom. Each interview was moderated by a provisionally registered psychologist undertaking Clinical Psychology training (IO), who directed the discussion through key questions. A senior clinician supervisor (FK) was present during the first two group interviews to assist with technical or clinical support.

The interview questions were designed using Krueger and Casey^32^ guidelines for structuring questioning routes (i.e. opening, key, and ending questions) and focus group/group interview questions (i.e. open-ended, succinct, and one-dimensional). Key questions were developed around three core topics: the impacts of the carer experience on their psychological wellbeing; experience accessing services and supports for their psychological wellbeing; and the kinds of supports participants would like for their psychological wellbeing (see Table 1). The term “psychological wellbeing” was used intentionally as a broad term to avoid limiting participants’ responses. When needed, prompts were offered to help participants consider psychological wellbeing in terms of their autonomy, personal growth, sense of purpose, environmental mastery, relationships, and self-acceptance,^33^ which has been defined similarly in previous studies with dementia carers.^34,35^

**Table 1.** Interview Schedule.

Impact on psychological wellbeing and mental health	Has your family member or loved one’s experience with bvFTD impacted your own psychological wellbeing? How?
	What has helped you manage these feelings?
	To what extent has it changed over time?
Services and support	What services or supports have you accessed to address your own psychological needs/wellbeing?
	Can you tell us more about: The focus and frequency of support, the most helpful aspects, potential improvements about the support, whether the support was sufficient?
	How did you find out about that service/support? Were there challenges or facilitators to accessing support?
	Has anyone here spoken to a psychologist or counsellor? If so, what was that experience like?
	For those of you that haven’t accessed any support, why might that be?
Needs	If we were to offer support for the psychological wellbeing of carers for people with bvFTD, what would you like this to address?
	How would you like these services to be delivered?
	If you had the opportunity to access only one psychological support for yourself, what would be most important/feasible?

*Note.* Key questions were delivered flexibly, and prompts, where necessary were provided. A more detailed interview schedule is included in the Supplementary Material.

### Data Analysis

The interviews were recorded (audio-visual and audio) and transcribed using the Zoom function. A member of the research team (IO) reviewed the transcripts and utilised the audio recordings and when necessary, audio-visual recordings, to identify and correct errors. Transcripts were uploaded to a qualitative computer data analysis program, NVivo (Release 1.7.1 for Mac), which was used as an organisational and data management tool to support the researchers in manually conducting thematic analysis;a qualitative method that aims to systematically organise large amounts of text into meaningful key themes.^31,36^ Thematic analysis was selected to support the study’s exploratory aims and bottom-up approach, given its flexibility as a qualitative method and its capacity to capture the breadth and richness of carers’ lived experience.^30,37^

Thematic analysis followed the 6-step process described by Braun and Clarke (2006)^29^ and additional guidelines on thematic analysis.^36-38^ The data was read through multiple times by IO, FK, and JT to allow for familiarity. IO manually coded each interview transcript on NVivo, and FK and JT then reviewed transcripts and initial coding to refine and identify additional codes. Coding was done inductively, and coding was set at the phrase level to accommodate the range by which participants described their experience. The research team met regularly throughout the interview, coding, and theme development process. Codes that captured related concepts and recurred across participants and interviews were constructed into an overarching theme and where applicable, subthemes. Themes were reviewed for their cohesiveness of meaning and representativeness of different participants’ experiences.

### Reflexivity

Field notes were taken by IO during each interview, and further reflections were documented by IO throughout the study to raise the research team’s sensitivity and reflexivity to the data. The research team comprised clinicians and researchers with expertise in clinical psychology and neuropsychology, and dementia. These professional backgrounds informed the design and interpretation of the data. Both FK and GW have extensive experience working with people with frontotemporal dementia and their carers, with an interest in post-diagnostic care. IO and JT are Clinical Psychologists and are interested in health psychology and applications of clinical psychological therapies in this context. The data and field notes were reviewed with FK and JT after each interview to determine whether changes to the interview questions were needed and discuss identified codes and themes. The research team met regularly to reflect on the interview process and content, and to discuss whether data saturation was achieved; that is, whether there was a diverse range of views, the identified themes were representative across participants and interviews, and there was no new information collected that could not already be captured by an identified code or theme.

## Results

### Demographics

Demographic characteristics of participants are displayed in Table 2. All participants identified as being the primary carer and spouse of someone with behavioural-variant frontotemporal dementia; two participants’ spouses had already died prior to the study. At the time of the interview, one participant reported that a diagnosis of behavioural-variant frontotemporal dementia, while likely, was still under investigation.

**Table 2.** Participant (carer) Demographics.

Demographic characteristics	
Gender (n)	Female (10)
	Male (2)
Age (years), mean (range)	68.33 (58 – 78)
Time caring (years), mean (range)	4.7 (1-8)
Employment (n, %)*	Part-time/Casual (2, 18%)
	Retired (6, 55%)
	Not working (3, 27%)
Geographical location (n, %)	Regional (4, 33%)
	Metropolitan (8, 67%)
Relationship status (n, %)	Married (11, 92%)
	Separated (1, 8%)
Living situation of the person living with bvFTD (n, %)	At home (6, 50%)
	In care (4, 33%)
	Deceased (2, 17%)

*Note*. Missing data for 1 carer.

### Themes

Five themes emerged: (1) *The job of being a carer*, (2) *The carer and person living with bvFTD as a unit: Reciprocal wellbeing & Care for the carers*, (3) *Feeling seen, Feeling heard, and Being connected*, (4) *The power of information*, and (5) *Meaning and purpose through caring*. Subthemes that emerged are discussed under each theme, with example quotes presented in Table 3.

**Table 3.** Example Quotes for Each Theme and Subtheme.

Summary of theme	Example quotations
**The job of being a carer**	
The role of bvFTD carer was likened to a new job, involving responsibilities as both carer and partner. Often this resulted in heightened stress and disruptions to participants’ lives outside of the carer role. Identified aspects of the job included carer guilt, advocacy, strength and resilience.	“*I had to almost command [his GP] to do these referrals…I was telling him what referrals I wanted rather than him talking to me…I basically became [my husband’s] GP*.” – PT11, FG4
	“*There’s a lot of guilt, I think, in handing somebody over to strangers and walking away, and the day that we took him [to the residential facility]—and to be honest with you, I didn’t discuss it with him really—I didn’t say, ‘is this what you want? You know, we’re going to take you to this place.’ I was at a point where I simply had no other option.*”– PT02, Int 1
	“*I’m a bit proud of the things I’ve managed to do. Does that sound strange? But I just thought wow, I actually managed that. We did alright, we kept [my partner] here, and he was very content*.” – PT05, FG1
**The carer and person living with bvFTD as a unit: Reciprocal ** **wellbeing & Care for the carers**	
The wellbeing of participants and the person living with bvFTD were interdependent, in both positive and negative ways, and so it was important to provide mutual care to both the person living with bvFTD and the carer.	Subtheme: Reciprocal wellbeing
	“*I tried to do things with him to make him happy. So, if he was happy, I was happy. My thing was him*.” – PT07, FG2
	“*I found that the apathy, the lack of empathy, all those sorts of behaviours really, really confronting and very distressing. From a man who was the exact opposite of all of those things… I was incredibly upset about all that. Didn’t understand what was going on.*” – PT11, FG4.
	Subtheme: Care for the carers
	“*Anything that is gonna help us to [look after our loved ones] or help us to feel better about ourselves while we’re doing that is important. Because ultimately it benefits the person with dementia to have someone there at the top of their game and doing the best job they can*.” – PT03, FG1
**Feeling seen, Feeling heard, and Being connected**	
Recognition and acknowledgement as bvFTD carers, feeling understood and believed, and being linked to social, emotional, and practical support networks significantly impacted participants’ psychological wellbeing, and influenced the extent to which psychological support was needed.	Subtheme: Feeling seen
	“*People just don’t understand there’s different kinds of dementias. And I still get that sense from [dementia group] that they sort of treat everything like it’s Alzheimer’s and if it’s not, it doesn’t fit in their little box, and you don’t get as much support*.” – PT02, FG3
	Subtheme: Feeling heard
	“*So probably my biggest stress [was his doctor] basically ignoring my pleads for what was going on with him…that battle gets very frustrating to say nobody knows him better than I do. And when I say something, you have to listen to me. Because I’m more worried about his best interest than anybody on the planet*.” – PT02, FG3
	Subtheme: Being connected
	“*I’m extremely fortunate in terms of my network of friends and family and so I haven’t been on my own through all this. I have had various people who’ve given me various kinds of support, so in that sense I haven’t sort of had to set my mind to thinking ‘oh gosh, there’s this big gaping hole in my life in terms of what I need*”. – PT10, Int 2
	*“But I live 40 km from the nearest town; it doesn’t have facilities or anything and there was nothing there for us, nowhere close. So I persevered and friends helped me get through that…I could just talk to them like a therapist and then they might give me some ideas. Go and have a drink. Go for a walk. We’re going to lunch.”* – PT07, FG2
	*“I think again, individual kind of hobbies and sports. Me, I like singing and dancing, and in the Filipino community we love to dance, we love karaoke, and we love singing, meeting in a group, and bringing food. Socialising is so important in Australia…there’s like longing to have someone to talk to and someone to listen to.”* – PT06, FG2
**The power of information**	
Increased information about bvFTD helped participants to do their “job” and manage the uncertainty about their future. Increased bvFTD knowledge in health professionals and awareness in the general population was important for addressing diagnostic delays, experiences of not being acknowledge and believed, and access to appropriate bvFTD carer support.	“*Knowledge is so important because it’s powerful. If you know and had strategies ahead of time to deal with what’s happening, you have a lot more confidence [in this] big world of unknown…. I can handle that because I had this knowledge. I know where to go. I know where to look …it’s knowledge so that I am prepared when these things happen, and I can feel like I’m doing my job*.” – PT12, FG4
	“*His GP says [to the person living with bvFTD] how’s your memory problem? I got a referral to a particular neurologist and this GP didn’t want to refer me. He said ‘no, just go to the memory clinic.” So I gave him a quick thing about how frontotemporal dementia isn’t necessarily about memory*.” – PT12, FG4
	“*I remember a certain amount of people being cross with this or that behaviour… So, anybody who knew him, and then was subject to that behaviour would just think he was being mean for some reason… So, I guess the more people know about frontotemporal dementia, they can look at his behaviour and think ‘Hmm. It’s almost as if [he] has frontotemporal dementia.’ Then that would be a bit helpful.*” – PT01, Int 1
**Meaning and purpose through caring**	
Using their carer knowledge and experience to help others, contribute to FTD research and participate in carer initiatives helped participants to find purpose and make meaning out of their experience.	“*I became very passionate about making the law better, because our experience with the local mental health team, who was trying to force me into putting my partner into permanent care, was really nothing less than traumatic… The [mental health] law has given me a long-time purpose, and FTD has given me a fairly long time purpose, because I’m really passionate about trying to raise awareness and make better for people who come after us. So that’s my coping strategy*.” – PT02, FG3
	“*I got invited to do a [FTD carer] talk to everybody. And I was really happy to be able to give back and do something that was occupying my mind. And we were about to set up a carer’s group where we’d have a number of people like myself who people could ring up and talk to… Knowing what I went through, that’s helping me to survive and keep going. If I help someone, I feel a lot better about myself*.” – PT07, FG2

### Theme 1: The Job of Being a Carer

Theme 1 “The job of being a carer”, emerged from participants’ experiences of facing increasing responsibilities as a partner and carer, with some participants referring to the carer role as this “new job.” No subthemes emerged.

As a partner or spouse, participants described taking greater responsibility over “*big ticket items*” (PT03, FG1) like managing personal, family and household affairs. Many carers referenced the younger age of onset of behavioural-variant frontotemporal dementia as contributing to a sense of overwhelm, as they “*thought we had plenty of years left to do that*” (PT05, FG1). As a result, participants were faced with a “*huge learning curve*” (PT05, FG1) that was often a source of great stress and anxiety particularly in the pre-diagnostic and diagnostic stages of the disease. Some participants also bore the responsibility of managing relationships within the person living with bvFTD’s network of family and friends. This included communicating the diagnosis and updates on the person living with bvFTD, and managing relationships with friends or children who were negatively impacted by their behaviour and personality changes. A few participants expressed a duty to “*stay strong*” (PT06, FG3) for friends and family by “*shielding*” (PT02, FG3) them from the reality of the impacts of behavioural-variant frontotemporal dementia. Several participants reported that at times, these relationship dynamics contributed to a sense of isolation and social disconnection.

As carers of someone living with behavioural-variant frontotemporal dementia, part of participants’ “*job*” was managing behavioural symptoms. This was perceived as stressful and anxiety-inducing as the behavioural symptoms often had implications for the financial security, safety, or relationships of the person living with bvFTD. The “*job*” also included carer duties like arranging appointments and accessing support for the person living with bvFTD. Some participants perceived these as more burdensome due to health professionals’ lack of knowledge about behavioural-variant frontotemporal dementia specifically; they described having to shoulder greater responsibility over the person living with bvFTD’s dementia care and advocate for their needs.

Some participants identified one of the most challenging aspects was managing carer guilt (e.g., when participants felt they were not doing their job well enough, when they increased professional service involvement, or when they transitioned the person living with bvFTD to residential care). Carer guilt also arose at points when participants felt angry, resentful, or frustrated about their situation. Nonetheless, despite the challenges of the job, some participants reported finding strength, resilience, and pride in their ability to cope and take care of the person living with bvFTD. This was conveyed in how participants grew accepting of their situation, made peace with the diagnosis, and then committed to doing their “*job*” as best as they can.

### Theme 2: The Carer and Person Living with bvFTD as a Unit: Reciprocal Wellbeing & Care for the Carers

Theme 2 “The carer and person living with bvFTD as a unit” emerged from participants’ reports that their wellbeing and needs were inevitably intertwined with those of the person living with bvFTD. Two sub-themes emerged: i. *reciprocal wellbeing,* describes how impacts to participants’ wellbeing were relational to the wellbeing of the person living with bvFTD and vice versa, and ii. *care for the carers,* describes the importance of offering support to carers who were impacted by the bvFTD diagnosis.

#### Reciprocal Wellbeing

All participants expressed how witnessing the continuous and complex changes in the person living with bvFTD’s personality, behaviour and functioning had negative impacts on their own psychological wellbeing. In the pre-diagnostic and diagnostic stages, these changes were experienced as “*confronting and very distressing*” (PT11, FG4) as participants found the person living with bvFTD behaving as “*the exact opposite*” (PT01, Int 1) to how they were prior to the onset of behavioural-variant frontotemporal dementia symptoms. Many participants also reported the person living with bvFTD becoming increasingly dependent on them due to loss of mobility or functional decline. In some participants, the increasing demands brought about physical exhaustion, despondence, and social isolation as they lost their independence and the “*life that I might have had, because life has become very singularly focused*” (PT03, FG1).

However, as participants gained a better understanding of behavioural-variant frontotemporal dementia as “*a condition that absolutely goes to the heart of an individual’s individuality*” (PT10, Int 2), the ongoing deterioration of the person living with bvFTD gave rise to a sense of grief. Grief was spoken about on multiple levels, as participants gradually lost the person living with bvFTD, their relationship, and the future they envisioned for themselves. Participants’ experience of grief was often associated with a disruption to retirement plans, physical intimacy, trust, and normalcy in things such as anniversaries, vacations, and meals. At times, these disruptions impacted participants’ capacity to care for the person living with bvFTD.

Conversely, participants expressed positive impacts on their wellbeing and greater capacity to cope when the person living with bvFTD was content and cared for. The majority of participants referred to positive experiences with services and health professionals of the person living with bvFTD (e.g. from a mix of publicly-funded research clinics, government-subsidised respite care, and hospital and community-based nursing or palliative care teams), such that they *“[couldn’t] separate one from the other. Any service or person that I had to visit for [the person living with bvFTD] invariably fed into my life in some way, usually more positively than negatively*” (PT05, FG1). Other participants described benefits to their own wellbeing when the person living with bvFTD spent quality time with family and friends, as this helped maintain a sense of normalcy and connection amidst the diagnosis of behavioural-variant frontotemporal dementia.

#### Care for the Carers

Participants emphasised that the person living with bvFTD’s wellbeing was also influenced by their own wellbeing. As such, several participants advocated for the provision of support specifically for carers. In providing carer-focused support, participants could have opportunities for rest, relaxation, and respite, which could then increase their capacity to look after the person living with bvFTD. Interestingly, not all participants expressed the need for formal carer support. This was the case for two participants who found that their partner’s symptoms and functioning were manageable at the time of the study or were not significantly distressed or impacted by their carer role. *“I haven’t felt the need to, I think that’s the number one thing…I haven’t gone looking for them [formal services] because it’s been good so far” (PT10, Int 2)*.*”* However, these participants acknowledged the utility of formal carer support for other carers and for when symptoms progress to be less manageable.

### Theme 3: Feeling Seen, Feeling Heard, and Being Connected

Theme 3 “Feeling seen, Feeling heard, and Being connected” describes how participants’ psychological wellbeing was impacted by how supported they felt at different points of their carer trajectory. The type of support is described in three subthemes: support through recognition and acknowledgement as carers (*feeling seen*), understanding and validation for their carer experience and concerns (*feeling heard*), and links to networks of social, emotional, and practical support (*being connected*).

#### Feeling Seen

A common perception among participants was that carers often got “*lost in the system*” (PT07, FG2). The majority of participants highlighted a lack of carer-specific services and of knowledge or behavioural-variant frontotemporal dementia-specific support offered to them by health professionals. Even when participants were aware of carer support, barriers such as proximity, limited flexibility, lack of government-subsidised funding, and complexities in navigating bureaucratic systems (e.g. NDIS, Centrelink, My Aged Care) often made accessing support “*extremely difficult and very haphazard*” (PT04, FG1) and “*almost impossible”* (PT01, Int 1). As a result, participants often felt isolated from support, that their role as carers was not recognised, and that the impact of behavioural-variant frontotemporal dementia on their own lives was considered unimportant.

In contrast, participants described feeling acknowledged when health professionals were knowledgeable about behavioural-variant frontotemporal dementia specifically or offered them carer-specific support. These included services for participants to be supported in their carer responsibilities, such as information days, frontotemporal dementia seminars and case managers, and services that allowed participants to focus on themselves such as carer days, support groups and counsellors. These services were offered through public and private hospital programs, government-funded social support systems, not-for-profit dementia organisations, publicly-funded research clinics, and private practices.

#### Feeling Heard

The experience of “*not being believed*” (PT12, FG4) was shared by most participants during the diagnostic period. Whereas participants noticed initial changes in the person living with bvFTD, others, including the person living with bvFTD themselves, did not recognise the symptoms of behavioural-variant frontotemporal dementia or mistook them as symptoms of relationship problems, psychological illness, or adjustment to retirement. As a result, participants described feeling discouraged, socially isolated, and even “*gaslit*” as others “*virtually [treated] you like you’re making [it up] and there’s nothing wrong*” (PT09, FG3). For some participants, the experience of not feeling heard continued post-diagnosis, particularly when coordinating with health professionals around the person living with bvFTD’s dementia care.

In contrast, participants shared how it was beneficial to have others listen to their experiences and concerns. Participants reported speaking to family or friends with similar experiences, and professionals (e.g., general practitioners, counsellor or psychologist through private practices, dementia organisations, and research clinics) who were knowledgeable about behavioural-variant frontotemporal dementia or displayed an effort to understand them. Peer support with other carers of people with frontotemporal dementia was also viewed as beneficial in helping participants to feel validated and understood; this was accessed via their personal networks, social network pages, and support groups run by hospitals and dementia organisations or research clinics. Peer support seemed particularly important in working through carer guilt: “*People [from the carer support group] say, ‘Hey, that’s pretty normal. You’re going to have days when you really feel down on yourselves… It just allows you to be human and gets you away from this notion that you have to be damn near perfect all the time*” (PT03, FG1).

#### Being Connected

Participants emphasised the importance of connecting to emotional, practical, and social support networks. Connecting to others for emotional support allowed participants to process feelings of anger, fear, and grief. Some participants engaged with informal supports (e.g., friends, family, local community), whereas others connected with more formal services including general dementia support groups, counsellors or psychologists (provided by hospital-run groups, private practices, and dementia organisations and research clinics). Whether participants engaged more in formal or informal support depended on factors including accessibility and proximity, participants’ capacity to cope or rely on their informal support system, and personal beliefs about accessing formal support: “*People told me I should call the [psychologist and counselling] service. In the end it almost made me feel like I was like being weak… I do have a really good informal support network. So, I just made a little more effort to get out of the house and try and connect with friends.*” (PT02, FG3). Participants also emphasised the value of having a social network. Through this network, participants could connect to a semblance of normalcy, leisure, and enjoyment, and have access to a wider range of experience outside the “*singular activity of caring for [the person living with bvFTD]* (PT02, FG1).” Some examples included meeting up with friends for regular meals, going to morning teas with other carers, engaging in hobbies, and spending quality time with family.

Lastly, having a network of practical support allowed participants to feel that they were not alone in their carer role. Practical support included nurses and external carers from hospital and community-based teams or private clinics, and family and friends who would share the carer responsibilities. It also included care coordinators or case managers (via government-funded social support systems) who could provide practical assistance with finances, navigating bureaucratic systems, and other personal affairs. Having people who could take over carer duties enabled participants to take a break, engage in their lives outside of the carer role (e.g. social activities, hobbies, self-development through work or study), and for two participants, stay in formal respite facilities for extended periods of time. In contrast, several participants described how difficulties outsourcing practical support for the person living with bvFTD made it infeasible for them to take a break or attend carer services by themselves.

### Theme 4: The Power of Information

Theme 4 “The power of information” captures how information and knowledge were most valuable in helping participants to manage the anxiety and uncertainty brought about by behavioural-variant frontotemporal dementia. No subthemes were identified.

Participants found that it was important for them to learn about behavioural-variant frontotemporal dementia so that they could better understand the person living with bvFTD and gain strategies to manage specific symptoms of behavioural-variant frontotemporal dementia. It was also important for participants to know which services and resources they needed for the person living with bvFTD at different stages of disease progression, and what carer supports were available. Participants obtained this information from a mix of sources: health professionals, information seminars and carer support groups run by dementia and research organisations, people within their personal network, and online carer forums. Many participants delved into their own research while on the road to diagnosis, or at points where they felt the information provided to them was insufficient.

Participants also emphasised the power of awareness of behavioural-variant frontotemporal dementia more specifically, as opposed to general information about dementia. Limited knowledge of health professionals was thought to contribute to diagnostic delays, experiences of not being believed, and confidence in accessing support appropriate for behavioural-variant frontotemporal dementia. For example, several participants were either referred to support geared toward Alzheimer’s disease or not offered support at all due to their partner’s diagnosis: *“...the geriatrician told us: so it looks like this is frontotemporal dementia, and there’s nothing more we can do for you. So all the best, and see you later.”* (PT12, FG4). Meanwhile, a lack of awareness in the general community of behavioural-variant frontotemporal dementia and its accompanying behavioural and personality changes was thought to contribute to disruptions in friendships and family dynamics, particularly when the diagnosis was still uncertain.

### Theme 5: Meaning and Purpose through Caring

Theme 5 “Meaning and purpose through caring” refers to participants’ ability to take ownership of their carer experience and find purpose in it, despite the different ways in which behavioural-variant frontotemporal dementia “*had turned life upside down”* (PT04, FG1). No subthemes emerged.

Many participants described utilising their knowledge and experience to help other people in their lives going through similar situations, and that this provided them with a sense of purpose. Participants also found that participating in frontotemporal dementia research, supporting carer initiatives, and advocating for awareness of behavioural-variant frontotemporal dementia allowed them to “*help the cause…and make better for people who come after us* (PT02, FG3)”—in doing so, participants were able to make meaning out of their carer experience. Participants found these opportunities through frontotemporal dementia research clinics and online carer forums, as well as within their own personal networks and lines of work or study.

### Relationship Between the 5 Themes

**Figure 1.**
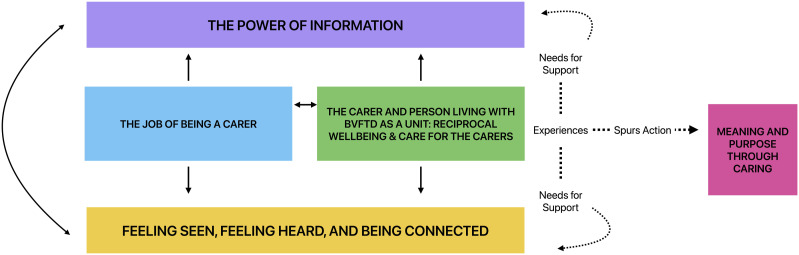
Proposed relationship between identified themes.

Participants’ overall experiences as carers and the impact these had on their psychological wellbeing were best captured by the themes “The job of being a carer” and “The carer and person living with bvFTD as a unit: Reciprocal wellbeing & Care for the carers”. For example, participants’ perception of whether support was provided to them as part of the care for the person living with bvFTD affected their ability to fulfill their “job” as carers. This in turn, influenced the wellbeing of the person living with bvFTD. Participants’ experiences then informed their needs for support, captured by the themes “The power of information” and “Feeling seen, Feeling heard, and Being connected.” For example, the increasing responsibilities participants faced in their “job” as carers made it important for participants to have comprehensive information about bvFTD and to feel acknowledged, understood, and connected to appropriate support networks. Lastly, from participants’ experiences and the gaps in support that they encountered, they identified the benefit of making meaning through and finding purpose whilst caring, as captured in the last theme. The proposed relationship between the themes is illustrated in Figure 1.

### Implications and Recommendations for Carer Support

Participants proposed concrete suggestions for supports for their psychological wellbeing, as well as means to address common barriers to support. Most of the information was shared in response to Part 3 of the focus groups/interviews (See Supplemental Material), where participants were asked about what kinds of support they would like/need for their psychosocial well-being. Some of the information also came through in Part 1 (impacts of carer experience on psychosocial wellbeing) and Part 2 (what supports have they accessed) of the interview (See Supplemental Material). The kinds of support participants talked about in Part 3 was informed by the impacts to their psychosocial wellbeing and their experience accessing support. These recommendations for support are summarised in Table 4.

**Table 4.** Participant Recommendations on Supports and Barriers to Address.

Recommendations for carer support
• A centralised website distributed between clinicians, dementia and research organizations, social networking groups and facilitated carer groups. This website can be co-developed by knowledgeable health professionals and carers, and include information on:
◦ The early signs and nature of disease progression
◦ Recommended strategies and person living with bvFTD-focused services for managing behavioural-variant frontotemporal dementia and its symptoms across different points of disease progression
◦ A directory of behavioural-variant frontotemporal dementia-informed health professionals and tailored carer supports (e.g. facilitated support groups, seminars, carer morning teas) in different localities
◦ Anecdotes and stories from carers
◦ An interactive forum wherein individuals can post questions and answers
• Opportunities for carers to share their experiences and receive emotional support for acceptance, grief, and carer guilt. Suggested avenues include individual peer support, facilitated carer support groups, or informed counsellors or psychologists.
• An organised peer-support directory of carers willing to be contacted to provide support to other carers
• A written resource co-developed by carers documenting their experiences, helpful strategies and advice for other carers
• Organised opportunities for leisure, creativity and social interaction amongst carers (e.g., dance classes, art workshops, book clubs)
• Development of supports as above, tailored for bereaved carers
• Supports to provide practical advice or assistance to fulfill carer responsibilities e.g.,
◦ Case managers to support carers in identifying and coordinating between necessary person living with bvFTD-focused services
◦ Practical support to navigate bureaucratic systems when accessing funding and support for the person living with bvFTD
◦ Support from an informed professional in managing financial, legal and household affairs
• Collaboration between carers, researchers and health practitioners in the design and development of services for carers
Addressing barriers to support
• Increased education and training for health professionals to better support carers and families
• Consolidating information on available carer services in different localities, and disseminating this information through health professionals, dementia organisations, social networking pages or a centralised website as above
• Increasing funding for support that is specifically for carers, rather than indirect support through the funding of the person living with bvFTD
• Options for online/remote access of carer services
• Developing a service or directory of dementia carers, support workers or nurses who can provide short-term (i.e. day, half-day) care for the person living with bvFTD

## Discussion

This qualitative study examined how caring for someone with behavioural-variant frontotemporal dementia impacts on carers’ psychological wellbeing, and carers’ experiences and needs in accessing support for themselves. Our findings revealed that caring for someone with behavioural-variant frontotemporal dementia substantially impacted on participants’ psychological wellbeing. While participants recognised that carer services for frontotemporal dementia or dementia more broadly (i.e. carer support networks, information seminars and counsellors) were helpful for their psychological wellbeing, participants experienced challenges in accessing and maintaining support, and these challenges discouraged participants from seeking further support. Support for the person with behavioural-variant frontotemporal dementia was emphasised as having reciprocal benefits to participants’ wellbeing, and increased awareness of behavioural-variant frontotemporal dementia as vital to the carer experience. Interestingly, many participants reported speaking with a psychologist or counsellor during their career trajectory through a dementia organisation, research clinic, or private practice. However, when participants were asked about the kinds of support they would like or need for their psychological wellbeing, they emphasised broader forms of practical, emotional, and social support as the most important, such as those accessed from respite and case management services, family, friends, and support groups. While psychologists and counsellors could also provide some of this support, formal psychological interventions (e.g. cognitive-behavioural therapy, acceptance-based interventions) were not identified by participants as a key unmet need. Nonetheless, this does not preclude the potential utility of specialised, targeted psychological interventions for this cohort. Development and evaluation of such interventions should consider the lived experiences and perspectives of carers. Moreover, emotional support, where needed, was more sought after from participants’ informal network of family, friends, and fellow carers. Lastly, a novel finding of this study was the importance of enabling carers to connect to their lives outside of the carer role and find purpose through their carer role, which is elaborated on below.

Participants in our study highlighted limitations in the availability and access to support specifically for carers. Recent changes to government-funded social support systems in Australia have shifted funding models such that carers access carer support through the funding allocated to the person living with bvFTD, rather than being provided directly to the carer.^39^ As a result, many carers lost access to support groups, counsellors, and case managers that were previously available through hospital programs or dementia organisations. These changes also limited access to community-based supports designed to provide social, recreational, and respite services for older people living at home.^39^ Surprisingly, practical and logistical challenges to accessing services, including navigating complex bureaucratic systems and limited information about available support, were major barriers identified in this study. Indeed, previous research suggests that system-related challenges like coordinating across providers and lack of transparency in services exacerbate the burden on carers.^6,40-42^ Our study builds on previous research by demonstrating that enhancing accessibility and flexibility of support, ensuring transparency in the support access process, and assisting carers in navigating bureaucratic systems is likely to benefit carers’ wellbeing.

A preference for supports to be tailored to carers of people living with behavioural-variant frontotemporal dementia over generic carer supports was reported, which aligns with prior work.^24,43^ In our study, several participants reported experiencing the lack of behavioural-variant frontotemporal dementia-specific support: they were offered services (i.e. support groups, respite services) more appropriate for older individuals with Alzheimer’s disease or felt support was not offered to them because their partner had behavioural-variant frontotemporal dementia instead of Alzheimer’s disease. Even some formal carer supports accessed by participants were tailored for carers of people with mixed frontotemporal dementia variants rather than behavioural-variant frontotemporal dementia specifically. Although general frontotemporal dementia support was perceived as helpful, our results suggest that generic “dementia programs” are less useful and desired than tailored support programs. These findings align with emerging research and protocols developing more tailored support programs for carers of people with rare forms of dementia, including behavioural-variant frontotemporal dementia.^44,45^

Importantly, lack of knowledge and information among clinicians about behavioural-variant frontotemporal dementia was a key issue. We found that limited awareness of behavioural-variant frontotemporal dementia in health professionals and the general community was believed to contribute to diagnostic delays, due to misinterpretation of symptoms of behavioural-variant frontotemporal dementia. This is consistent with prior studies that have reported experiences of behavioural-variant frontotemporal dementia and frontotemporal dementia carers feeling as though their concerns about the person living with frontotemporal dementia were dismissed in the pre-diagnostic stage.^6,15,18,22,23^ Furthermore, carers of people with frontotemporal dementia more generally are less satisfied with the dementia information provided to them compared to carers of people with Alzheimer’s disease,^46,47^ and healthcare providers often lack the necessary training to provide ongoing support for carers to manage frontotemporal dementia.^21,24,48^ Thus, targeted training for healthcare providers about diagnosis and care for people living with behavioural-variant frontotemporal dementia across the disease trajectory is an essential area for improvement.

Surprisingly, formal psychological interventions (e.g., cognitive behavioural therapy) were not identified as an area of need. This may reflect the fact that distress, anxiety, and burden may be a normal response as carers adjust to profound changes in their loved one, relationship, and lives. Rather than targeted psychological treatment, interventions offering broader psychological support for carers of people living with behavioural-variant frontotemporal dementia to adapt to their situation may be more suited to their needs.^25,49,50^ Indeed, interventions for dementia carers typically take a psychoeducational or multi-component approach, integrating educational and skill-building components with psychotherapeutic techniques from cognitive behaviour therapy and acceptance- and mindfulness-based therapies.^26-28^ Programs such as the European RHAPSODY project have shown that web-based psychoeducation interventions can provide accessible and feasible support for carers of people with young-onset dementia across multiple countries, reducing carer stress and negative emotional responses.^51-54^ Similarly, the Partner in Balance program in the Netherlands combines web-based psychoeducational training with coaching from allied health professionals, including psychologists, and has been found to improve self-efficacy and emotional wellbeing among carers.^55^ To date, such programs have not been adapted or widely implemented in an Australian context. However, collectively, these findings suggest that psychoeducational or multi-component interventions can offer meaningful support for carers and may serve to complement formal psychological interventions.

Of relevance here, we specifically excluded participants with acute or serious mental health difficulties. Thus, there may be a subset of carers who will benefit from formal psychological interventions, particularly the subset of carers of people living with behavioural-variant frontotemporal dementia who endorse clinically significant mental health difficulties.^7,12,13^ Our cohort was also somewhat unusual in that several participants had access to mental health support through their personal networks or professional training. Consequently, who is likely to benefit from formal psychological support tailored for behavioural-variant frontotemporal dementia and what this intervention should encapsulate, warrants further quantitative investigation.

Our research found that enabling carers to find purpose within and reconnect to life outside of their carer role can have positive effects on psychological wellbeing. This includes finding meaning in sharing their carer knowledge and contributing to frontotemporal dementia research or carer initiatives. Indeed, many participants expressed that participating in this study allowed them to connect with fellow carers of people living with behavioural-variant frontotemporal dementia, identify common support needs, and propose carer supports and ways to address barriers to these. Our findings illustrate that finding fulfilment in the carer role, strengthening social connections, and actively engaging in services that help others in similar situations can facilitate resilience and optimise positive carer experiences. Moreover, our results indicated that engaging in social activities, hobbies, and self-development through work or study opportunities allowed participants to experience moments of normalcy and reclaim aspects of their lives; activities which are often sacrificed to fulfil carer responsibilities. Our findings suggest that empowering carers with agency and purpose within their carer experience can be a vital support amidst the lack of control over the changes in the person living with bvFTD and their own lives.

Although our study provides important insights into the carer experience of behavioural-variant frontotemporal dementia, some limitations warrant consideration. First, our study included a small sample of carers, all of whom were well-educated, had no acute or serious mental health concerns, and were recruited from a frontotemporal dementia research organisation providing multidisciplinary care. Despite being relatively well-supported, participants nevertheless expressed needs for support and experienced barriers to services. It is important for future studies to examine the support needs in larger groups of carers and of carer who are potentially more vulnerable, such as those from lower socioeconomic backgrounds, living in regional/remote areas, and experiencing significant mental health difficulties. Second, participants were spousal carers, most of whom were female and further along their carer trajectory. Future studies exploring differences in support needs amongst carers of different genders, non-spousal carers, and carers at different stages of the carer trajectory, including pre-diagnosis and bereavement will be important to complement our findings. Lastly, our study was conducted in the Australian context with carers who were all English-speaking. It is likely that similar carer experiences arise in the broader population of people living with behavioural-variant frontotemporal dementia, particularly in other Western cultures like United States or United Kingdom. Nonetheless, it would be valuable to examine the experience of carers from culturally and linguistically diverse backgrounds, such as Eastern cultures, where carer responsibilities may be shared between immediate and extended family members, and carers may rely more on informal than on professional support.

## Conclusion

This study highlights the positive and negative impacts of the carer experience on carers’ psychological wellbeing and emphasises the need for increased carer-focused support in the context of behavioural-variant frontotemporal dementia. Although carers of people living with behavioural-variant frontotemporal dementia identified services that were helpful for their wellbeing, difficulties accessing this support often deterred carers or made it difficult for them to receive support. This bears implications for future carer services to address common barriers to accessibility and flexibility of support. Lastly, this study shows that carers of people living with behavioural-variant frontotemporal dementia have capacity to find purpose and make meaning in their carer role, by using their knowledge to help others and contribute to the broader frontotemporal dementia cause. Our findings provide insights for enhancing collaboration between researchers, practitioners, and carers when developing and improving support for this population.

## Supplemental Material

Supplemental Material - The Impact of Caring on Psychological Wellbeing: A Qualitative Study in Carers of People Living With Behavioural-Variant Frontotemporal Dementia Supplemental Material for The Impact of Caring on Psychological Wellbeing: A Qualitative Study in Carers of People Living With Behavioural-Variant Frontotemporal Dementia by Isabella Maria Beatrice M. Ocampo, Jemma Todd, Grace Wei & Fiona Kumfor

## Data Availability

The data that support the findings of this study are not publicly available due to privacy reasons but are available from the corresponding author upon reasonable request.*
