# Supplementary material for: The Impact of Caring on Psychological Wellbeing: A Qualitative Study in Carers of People Living With Behavioural-Variant Frontotemporal Dementia
Source: J Geriatr Psychiatry Neurol. 2025 Nov 21;39(4):534–48. doi: 10.1177/08919887251401262 (PMC13103344; doi:10.1177/08919887251401262)
Supplement: Supplemental Material - The Impact of Caring on Psychological Wellbeing: A Qualitative Study in Carers of People Living With Behavioural-Variant Frontotemporal Dementia [file sj-pdf-1-jgp-10.1177_08919887251401262.pdf]

Supplementary Material

Interview Questioning Route		
**Section**	**Questioning Route**	**Time**
**Opening**	I'd like you to introduce yourself and tell us:<br>· How long have you been caring for someone living with bvFTD?<br>· Are they living with you at the moment?<br>· Just briefly, what your experience caring for someone living with bvFTD has been like?	10 minutes
**Key questions**	See below the interview schedule	70 minutes
**Ending**	· Summary and summary question, "The purpose of our study is to…. (describe purpose and summarise discussion) … Is there anything else you would like to add?"<br>· All things considered question, "Of all the things we talked about, what to you is the most important?"	5 minutes

Interview Sample Questions	
**Topic guide**	Examples of key questions (dot bullets) and prompts, if needed (open bullets)<br>(*Questions will be delivered flexibly; however, the information sought will be maintained)*
**1. Impact on psychological wellbeing**	· Has your family member or loved one's experience with bvFTD impacted your own psychological wellbeing? How?<br>o To what extent has it affected your (mood, feelings of stress, feelings of worry, sense of autonomy, sense of purpose)?<br>o Has your experience caring for someone living with bvFTD changed the way you think about (yourself, them, your future, others, the world around you)? If so, how?<br>o What is it about your caregiving experience that you find is making this change? (Prompts: relationship, role changes, grief, symptom management, acceptance/denial of diagnosis)<br>· What has helped you manage these feelings?<br>· To what extent has it changed over time?<br>o When did it start to affect your own psychological health or wellbeing? Can you talk more about this?<br>o When were these impacts most intense?<br>o Do you find that these impacts were getting better or worse over time?

**2. Psychological services and support**	· What services or supports have you accessed to address your own psychological needs/wellbeing?<br>· Specific examples to prompt responses:<br>o Have you relied on informal supports?<br>§ Have you spoken to your family or friends?<br>§ Have you sought online resources?<br>o Have you accessed more formal supports?<br>§ Have you seen a GP or health practitioner with these concerns?<br>§ Have you participated in support groups?<br>§ Have you seen a psychologist or a counsellor?<br>· Can you tell us more about that?<br>o What was the focus?<br>o How often did you receive this support?<br>o What was most helpful about the support?<br>o Did you feel that the support provided was sufficient?<br>o If you could change or improve one thing from that service, what would that be?<br>o Was there one specific service that you found particularly helpful? Why?<br>· How did you find out about that service/support?<br>o Were there any challenges finding services?<br>o What helped you access that support?<br>o Do you feel that there are sufficient supports or services, or information about these available for you as a carer?<br>· Specific questions about formal psychological intervention:<br>o Has anyone here spoken to a psychologist or a counsellor?<br>o Have you thought about it?<br>o How would you go about seeing a psychologist?<br>o Is it important that they would have experience or knowledge about dementia?<br>o What do you think a clinical psychologist does?<br>· For those of you that haven’t accessed any support, why might that be?<br>o Would you want / have wanted to receive support for your psychological needs?<br>o What has gotten in the way of you getting support?<br>· What is something that might help you access support?

**3. Needs in terms of psychological supports**	· If we were to offer support for the psychological wellbeing of carers for people living with bvFTD, what would you like this to address?<br>· Prompts about content or focus of services:<br>o Some work with carers mention stress and anxiety/low mood. Do you think that these would be important to address?<br>o Would it be helpful to manage emotions such as frustration or anger/feelings of loss or grief?<br>o Would it be helpful to manage changes in your role or relationships?<br>o Would it be important to address self-care and personal boundaries?<br>o Would it be important to provide education or skills specific to bvFTD?<br>· How would you like these services to be delivered?<br>o Would you prefer for them to be delivered in-person or online?<br>o Would you prefer them to be in a group or individual format?<br>o Would you prefer to them to be self-guided or therapist-guided?<br>o How often and for how long would you like to access these services? (What might get in the way of you accessing these services?)<br>o Would you like specific topics to be discussed then have free time to chat?<br>o +why<br>· If you had the opportunity to access only one support for your psychological wellbeing needs, what would be most important/feasible?
**Probes**	· Can you explain further.<br>· Can you give us an example?<br>· Tell us more.<br>· Is there anything else?<br>· Can you describe it a bit more?<br>· Please describe what you mean.<br>· What experiences have you had that make you feel that way?<br>· Tell me what you mean when you say X.<br>· If I were in the room when this happened, what would I see?

**Giving license to express differing points of view**	· Does anyone see it differently?<br>· Has anyone had a different experience?<br>· Are there other points of view?<br>· No one has mentioned it here, but in other groups we have heard X. What’s your reaction to that?<br>· In our discussion we have not talked about X, and I was wondering if that is important to you?
